# Healthy Cities, A comprehensive dataset for environmental determinants of health in England cities

**DOI:** 10.1038/s41597-023-02060-y

**Published:** 2023-03-25

**Authors:** Zhenyu Han, Tong Xia, Yanxin Xi, Yong Li

**Affiliations:** 1 Beijing National Research Center for Information Science and Technology (BNRist), Beijing, P. R. China; 2 Department of Electronic Engineering, Tsinghua University, Beijing, P. R. China; 3 Department of Computer Science and Technology, University of Cambridge, Cambridge, UK; 4 Department of Computer Science, University of Helsinki, Helsinki, Finland

**Keywords:** Environmental impact, Public health, Risk factors

## Abstract

This paper presents a fine-grained and multi-sourced dataset for environmental determinants of health collected from England cities. We provide health outcomes of citizens covering physical health (COVID-19 cases, asthma medication expenditure, etc.), mental health (psychological medication expenditure), and life expectancy estimations. We present the corresponding environmental determinants from four perspectives, including basic statistics (population, area, etc.), behavioural environment (availability of tobacco, health-care services, etc.), built environment (road density, street view features, etc.), and natural environment (air quality, temperature, etc.). To reveal regional differences, we extract and integrate massive environment and health indicators from heterogeneous sources into two unified spatial scales, i.e., at the middle layer super output area (MSOA) and the city level, via big data processing and deep learning. Our data holds great promise for diverse audiences, such as public health researchers and urban designers, to further unveil the environmental determinants of health and design methodology for a healthy, sustainable city.

## Background & Summary

As urbanization progresses, millions of people have flocked to cities. It is reported that nowadays more than 55% of the world’s population lives in urban areas. A good environment is crucial to healthy and sustainable cities^1–4^, yet, air pollution^5^, deteriorating climates^6–8^, unavailability of public green spaces^9–12^, inadequate water, sanitation and hygiene^13^ are continuously threatening the citizens’ health. As an example, the poor air quality in the UK caused nearly 29,000 deaths and an associated loss of population life of 340,000 life year lost in 2008^14^. Besides, unhealthy lifestyle caused by easy access to alcohol and the lack of green or blue spaces in cities also yields notably negative effects on citizens’ physical and mental health^15,16^. Collectively, non-communicable diseases account for nearly 70% of global deaths each year before the COVID-19 outbreak^1,17^. To achieve the United Nations’ Sustainable Development Goals to “make cities and human settlements inclusive, safe, resilient and sustainable”, and “ensure healthy lives and promote well-being for all at all ages” by 2030^18^, in-depth understanding of the correlation between city environment and public health towards better urban planning and retrofit is of critical importance.

**Fig. 1:**
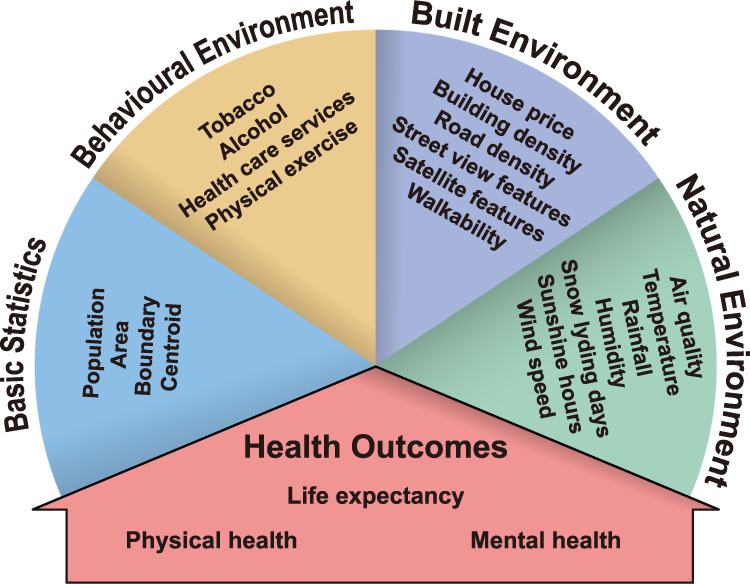
Schematic overview of the produced dataset.

However, a fine-grained and multi-sourced dataset covering heterogeneous environmental determinants of health that can support such studies is lacking. Previous publicly available data usually focus on specific environmental features, such as air pollution^19–21^, tobacco and alcohol accessibility^22^, or spatial distribution of health services^23^, which are scattered in different countries with varying spatial resolution. For copyright-protected databases such as UK Biobank^24^, much effort is still needed to merge the heterogeneous data. The scattered, messy-formatted data significantly increase the cost of research communities, where researchers have to do repetitive works to leverage these data. The high cost of scientific research has incurred public criticism, increasing the tension situation between research communities and taxpayers^25^. To bridge the data gap between the urban environment and the health outcome of citizens and the social gap between data sources and researchers, we present a comprehensive fine-grained health dataset of 1039 MSOAs in 29 England cities from 2019 to 2022. The topology of the dataset is illustrated in Fig. 1. It consists of two major components: the health outcomes of citizens and the corresponding environmental determinants. For the health outcomes of citizens, we consider the macroscopic life expectancy and the microscopic expenditures of several non-communicable physical and mental diseases. Since the outbreak of COVID-19 at the end of 2019, it has become the most representative communicable disease sweeping the whole world. Thus, we collect fine-grained COVID-19 cases to demonstrate the resilience of cities for pandemics^26,27^. For the environmental determinants, we adopt a hierarchical view from behavioural factors to natural environments^2,4^, where the recent advances in deep learning technology and big data processing provide the valuable opportunity to extract environmental determinants of health from heterogeneous data sources such as the road network, street view images and prescription records. Different from previous studies, we provide a unified comprehensive dataset to unmask the border picture of healthy cities.

Overall, this study aims to minimize the social costs to collect and generate fine-grained environmental determinants of health in urban spaces for both public health researchers and urban designers, who might not have the experience to process such heterogeneous big data. Providing a unified dataset and disclosing the data collection and generation processes promote the knowledge discovery in a cost-efficient manner, where the underlying higher-order linkages between multiple environmental factors with diseases can be further investigated through the provided data, and the derived urban patterns can also serve as indicators that shed light on the design of healthy, sustainable cities.

## Methods

**Fig. 2:**
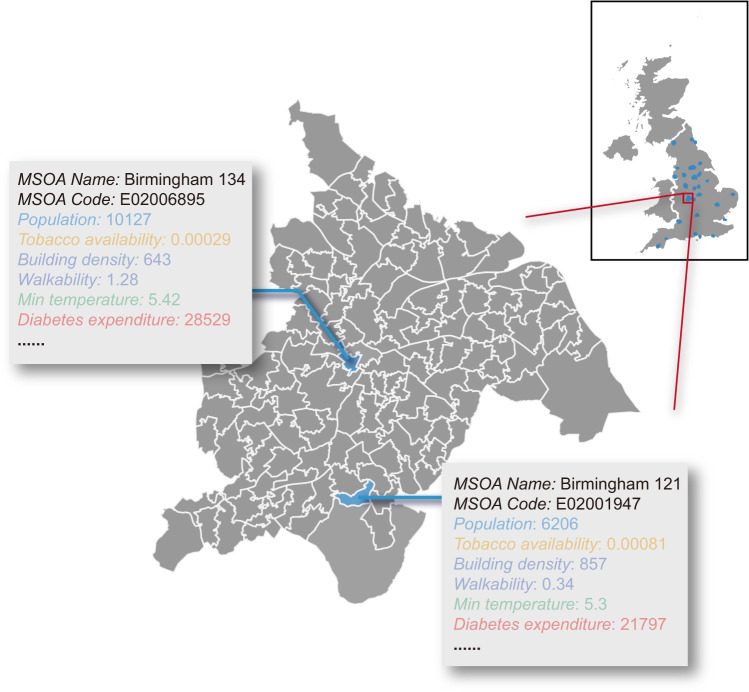
Example of data records in MSOA of Birmingham city. The color represents data category the record belongs to. For time series data, we showcase the first values.

Environmental determinants of health refer to regional, national, and local environmental factors that influence human physical, chemical, and biological health, and all related behaviours. To ensure the comprehensive coverage of various environmental factors, we select basic, behavioural, built, and natural environment descriptors (see Fig. 1 for details). The generation of the target dataset requires heterogeneous data collection, processing, and aggregation, which transforms the input data sources in Table 1 to the unified format illustrated in Fig. 2. We first introduce the determination of geographical units for the target dataset, then discuss the detailed generation process of each subsection of the dataset in Fig. 1.

**Table 1:** Information of input datasets.

Name	Destination Category	Data Type	Spatial Resolution	Time Period	Source
City list	General	Tabular	City level	2022	UK government^28^
MSOA-City lookup table	General	Tabular	MSOA level	MSOA 2011, City 2015	ONS Geography^31^
Postcode-MSOA lookup table	General	Tabular	Postcode level	2021	ONS Geography^32^
City boundary	Basic statistics	Polygon	City level, 50 m generalised	2015	ONS Geography^29^
MSOA boundary	Basic statistics	Polygon	MSOA level, 20 m generalised	2011	ONS Geography^39^
City area	Basic statistics	Tabular	City level	2015	ONS Geography^29^
MSOA area	Basic statistics	Tabular	MSOA level	2011	ONS Geography^39^
MSOA population	Basic statistics	Tabular	MSOA level	2020	ONS^38^
POI data	Behavioural environment	Tabular, point	Point level	2022	SafeGraph^43^
Road network	Built environment	Polygon	Point level	2022	OpenStreetMap Foundation & Contributors^50^
Building	Built environment	Polygon	Point level	2022	OpenStreetMap Foundation & Contributors^50^
Median house price in MSOA	Built environment	Tabular, time series	MSOA level	2019/03-2022-03, each 3 months	ONS^46^
Median house price in city	Built environment	Tabular, time series	City level	2019/03-2022-03, each 3 months	ONS^48^
Mean house price in MSOA	Built environment	Tabular, time series	MSOA level	2019/03-2022-03, each 3 months	ONS^47^
Mean house price in city	Built environment	Tabular, time series	City level	2019/03-2022-03, each 3 months	ONS^49^
Street view image	Built environment	Image	Point level	2022	Google Map^52^
Satellite image	Built environment	Image	MSOA level, 0.6 m pixel resolution	2022	Esri World Imagery^58^
Air quality	Natural environment	Time series	City level	2019/01/01-2022/08/31, daily	UK Air^66^
Weather data	Natural environment	Time series	1 km × 1 km grid (for MSOA)	2019/01/01-2021/12/31, daily	Met Office^74^
Weather data	Natural environment	Time series	12 km × 12 km grid (for city)	2019/01/01-2021/12/31, daily	Met Office^74^
Prescribing records	Health outcomes	Tabular, time series	Postcode level	2019/01-2022/08, monthly	NHS^34^
COVID-19 data	Health outcomes	Time series	MSOA level	2019/01/01-2022/08/31, daily	UK government^37^
Life expectancy	Health outcomes	Tabular	MSOA level	2015	ONS^33^

### Determining the geographical units

We select the city-of-interests according to the honour list of city status by the UK government^28^ and the Office for National Statistics (ONS) Geography definition of major towns and cities^29^, which captures the high status from both the cultural and economic perspectives. We further filter the cities with administrative power as lower tier local authorities (LTLAs), combining which we acquire 29 representative cities in England (see Table 2 for details).

**Table 2:** City-of-interests in our dataset.

City Name				
Birmingham	Bradford	Brighton & Hove	Bristol	Cambridge
Carlisle	Coventry	Derby	Doncaster	Exeter
Leeds	Leicester	Lincoln	Liverpool	Manchester
Newcastle-upon-Tyne	Norwich	Nottingham	Oxford	Plymouth
Portsmouth	Preston	Salford	Sheffield	Southampton
Southend-on-Sea	Stoke on Trent	Sunderland	York	

Datasets from heterogeneous sources often have different geographies: administrative geography, census geographies, postal geography, etc. A unified, fine-grained unit is of great importance to merge these data and unmask the relationship between environmental factors and their health outcomes, so as to support region-level comparisons^30^. Therefore, we select middle layer super output areas (MSOAs) as the main geographical unit in our study, which is a fine-grained census division that has a mean population of around 7200. As an illustrative example, we visualize the MSOAs of Birmingham city with valid data records in Fig. 2. As a more aggregated point-of-view, we also provide city-level aggregations in our dataset.

To merge collected data in different geographies, we collect MSOA-city lookup table^31^ and postcode-MSOA lookup table^32^ from the ONS Geography. By filtering and merging the collected lookup tables according to the city list, we generate a unified geography lookup table as shown in Table 3, which contains 1039 MSOAs. Those identified MSOAs are referred to as the minimum spatial units for our following data processing from all sources, which is used in the following generation procedures to merge the data.

**Table 3:** Example of essential information of geography lookup table for the produced dataset.

City Name	City Code	MSOA Name	MSOA Code	Post Code
Birmingham	J01000007	Birmingham 008	E02001834	B43 7DS
		Birmingham 008	E02001834	B43 7DT
		Birmingham 008	E02001834	…
		Birmingham 011	E02001837	B44 0AW
		Birmingham 011	E02001837	B44 0BB
		…	…	…
Bradford	J01000013	Bradford 017	E02001834	B43 7DS
		Bradford 017	E02001834	B43 7DT
		Bradford 017	E02001834	…
		Bradford 019	E02002201	BD100BA
		Bradford 019	E02002201	BD100BB
		…	…	…
…	…	…	…	…

### Processing of health outcomes data

We formulate the health outcome of citizens for each region from three aspects: life expectancy, physical health, and mental health. For life expectancy data, we collect gender-specific life expectancy and healthy life expectancy in MSOA level from ONS^33^, then filter the regions according to the geography lookup table described in Table 3. For physical health, we consider 6 common non-communicable diseases in cities: asthma, cancer, dementia, diabetes, hyperlipidemia, hypertension and obesity. For mental health, we mainly consider depression, psychosis and related disorders in cities. To accurately assess the severity of these diseases, we collect fine-grained prescribing data from the National Health Service (NHS) Business Services Authority^34^, which serves as an informative data source to estimate the health status of citizens. It contains the drug code, drug quantity, and corresponding expenditure for each practice such as a general practitioner (GP), out-of-Hours service, or a hospital department. Specifically, we focus on expenditure records since they can be used to comprehensively evaluate the severity of diseases across different drugs. Considering the large quantity of the data, we use the Open Data Portal Application Programming Interface (API)^35^ to query the required information. We filter their corresponding drug codes for physical health and mental health through the British National Formulary (BNF)^36^. Then we generate the corresponding structured query language (SQL) request through the API to acquire the aggregated actual cost data of these diseases in the postcode level. Since the outbreak of SARS-CoV-2 virus at the end of 2019, COVID-19 has become the most influential communicable disease in urban spaces. We also consider COVID-19 as a representative communicable disease affecting the physical health. For the COVID-19 data, we collect the MSOA level time series from the UK government^37^, which contains the number of new cases within rolling 7-day periods. During the post process, we merge them into MSOA and city level according to the geography lookup table.

### Processing of basic statistics data

The basic statistics data include the population, area, boundary and centroid of selected regions, providing essential information to understand the composition of urban spaces. Specifically, we collect the latest estimates of the usual resident population for MSOA level^38^, which is in mid-2020. We filter the population numbers of selected MSOAs^38^ and aggregate them to obtain the city population according to the geography lookup table. The up-to-date city boundary is defined in 2015^29^, which corresponds to the census result of 2011. Thus, we collect the geographical boundary^39,40^ and the geographical lookup table^31,32^ of MSOA in their 2011 definition. We adopt the generalized boundary within 20 *m* error range in our dataset, which strikes a good balance between accuracy and data size. For the boundary data, we filter the MSOA boundary^39^ and city boundary^29^ accordingly, and save the polygons in GeoJSON format with the corresponding MSOA codes and city codes. We preserve the original coordinate system of WGS84 in the resulting files. The above boundary data contain the area information of each region, where we modify the data unit into *km*^2^ level. According to the population data and area data, we calculate the population density of each MSOA and city in our dataset. For the centroid data, we use the Python packet *shapely* to calculate the geometric centroids according to the above boundary of cities and MSOAs.

### Processing of behaviour environment data

The venues in cities affect the behaviour of citizens in a subtle way, where researchers have demonstrated strong evidence that the availability of tobacco & alcohol^22^, open green spaces^9,10,41^, and medical resources^42^ affect the health outcomes. Here, we focus on the availability of tobacco, alcohol, physical exercise, health care services in a neighbourhood through point-of-interest (POI) data as important health-related behaviour factors. Specifically, we collect the SafeGraph Places Data Schema^43^, which contains more than 1.5 million records for the whole UK. We filter the POIs by their categories, which are in North American Industry Classification System (NAICS)^44^ 2017 version. NAICS is a classification system developed by the US Census Bureau, which uses a numeric code up to 6 digits in length to hierarchically classify different venues. For tobacco availability, we filter the POIs with NAICS categories of *Tobacco Stores* and *Grocery Stores*. We also calculate alcohol availability by *Drinking Places*, *Beer, Wine, Liquor Stores*, and *Grocery Stores*. For physical exercise availability, we consider *Fitness and Recreational Sports Centers, Nature Parks and Other Similar Institutions*. For health care services availability, we consider *Health and Personal Care Stores, Ambulatory Health Care Services, Hospital, Nursing and Residential Care Facilities*. Finally, we calculate the availability indicators by the fraction of corresponding POI numbers and region population.

### Processing of built environment data

Urban built environment, as an important determinant of health, shapes citizens’ physical activity and mental well-being^45^. In this study, we incorporate house price, building density, road network density, street view features, satellite features, and walkability to jointly describe the built environment of urban spaces.

We collect the median and mean house price data from ONS^46–49^, which include seasonally time series of MSOA level house prices from 1995 until now for both newly built and existing dwellings. It contains common house types such as detached houses, semi-detached houses, terraced houses, flats and manisonettes. Here, we extract the general indicator containing all sales and all house types for the selected regions in our study.

We collect the building information and road networks from OpenStreetMap^50^. To export large-scale map data, we use the bulk download service provided by Geofabrik^51^. We manually download the minimal subregion files that contain the city-of-interests, and use the Python packet *pyrosm* to extract the building information and road networks in interested cities and MSOAs by specifying corresponding boundary polygons. We count the number of buildings in each region, and calculate the building density by dividing it by the area size. For the road network, we filter the driving network, cycling network and walking network accordingly, and calculate the road density indicator by the ratio of total road length and the area size.

The availability of street view imagery provided by map platforms such as Google^52^ enables a new angle to observe and analyse the urban environment for the health outcomes for every citizen^53,54^. For the street view image data, we sample the urban spaces into 100 *m* × 100 *m* grids and download the 360° images from Google Map^52^, which generates 784 thousand images. With the recent advantages of deep learning technology, automatic feature extraction for large-scale image data is possible. In our study, we adopt the state-of-the-art semantic segmentation model ViT-Adapter^55^ based on vision transformer technology to automatically infer the objects in the street view images, which provides high-accuracy pixel-level classification to the input images. Specifically, we use the official implementation^56^ provided by the authors trained on Cityscapes dataset^57^ for our street view images. It recognizes 19 different objects in the image, which are shown in Table 4. We calculate the pixel-level percentage of each objects, and aggregate them in the MSOA and city level to capture the visual semantics of neighbourhood features.

**Table 4:** Recognized objects for street view and satellite view images.

**Street view images**	road, sidewalk, building, wall, fence, pole, traffic light, traffic sign, vegetation, terrain, sky, person, rider, car, truck, bus, train, motorcycle, bicycle
**Satellite view images**	building, road, water, barren, forest, agriculture, background

The satellite view imagery is obtained from Esri World Imagery^58^ according to the method described in^59^ and its corresponding code implementation^60^. Specifically, we collect 0.6 *m* resolution satellite image data tiles covering all the city-of-interests. Then we train the ViT-Adapter^55^ model on LoveDA dataset^61^ to extract the 7 labeled objects as features from the collected satellite images. Like the street view images, we aggregate the inference result images according to the MSOA and city boundaries, and calculate the pixel-level percentage of each annotated object.

Walkability is a long-standing indicator in the field of urban planning, which evaluates the mixed-use of amenities to quantify how walking-friendly a neighbourhood is^62^. In this study, we focus on the health benefit of walkability according to^30^, which defines walkability as the average z-score of population density, intersection density and a daily living score. We calculate the intersection density through the above OpenStreetMap walking road network data, where we use Python packet *shapely* to determine whether two roads have any intersection. We summarize the number of intersections in each region, and divide by the corresponding area size as the intersection density. For the daily living score, we consider the density of daily living POIs in each region. According to^30^, we define daily living POIs in the following categories: *Grocery Stores, Nature Parks and Other Similar Institutions, Air Transportation, Rail Transportation, Water Transportation, Transit and Ground Passenger Transportation*, and calculate the daily living score by dividing the total number of these POIs with the area size. We normalize the above three indicators according to the following equation

1 $${Z}_{\ast }=\frac{{x}_{\ast }-{\mu }_{\ast }}{{\sigma }_{\ast }},$$

where x_*_ could be the population density, intersection density or daily living score, and *μ*, *σ* are the mean and standard variation of x_*_. Finally, we derive the walkability score by taking the average of normalized indicators.

### Processing of natural environment data

Exposure to polluted air is considered a major health challenge for citizens^63–65^. The air quality data is obtained from UK Air^66^, which is organized by the Department for Environment Food & Rural Affairs (DEFRA). We focus on the Automatic Urban and Rural (AURN) monitoring network, which is the UK’s largest automatic monitoring network for common air pollutants. Specifically, we collect the daily mean records of nitrogen oxides as nitrogen dioxide, PM2.5, and PM10 particulate matter as the air pollution indicators in our dataset. The collected data are available at the station level. We manually select the stations and the corresponding pollution data according to the interactive map^67^ and station information^68^. Specifically, for cities with multiple stations, we preserve all the observations in our data.

Climate issue ties tightly with the well-being of all the people^69–71^. Recently, new evidence shows that worsening climate is correlated with a variety of health outcomes, including insufficient nutrition, pandemic outbreaks, and increasing of anxiety and depression^72,73^. To evaluate how the changing weather affects the health outcome in each region, we collect the weather data from HadUK-Grid maintained by Met Office^74^, which is a collection of gridded climate variables in high spatial resolution. We collect temperature, precipitation, relative humidity, sunshine duration, snow lying days, and wind speed as the weather features. During the post process, we align the grid data of weather into MSOA and city level. Specifically, we use Python packet *h5netcdf* to read the weather data, which are provided in NetCDF format. Then we calculate the distance between the gridded data point with the geometric centre of each region by Python packet *haversine*, and match the nearest one as the target. Considering the size of MSOA and cities, we use 1 *km* × 1 *km* resolution data to match each MSOA, and 12 *km* × 12 *km* data to match each city.

## Data Records

The produced dataset is publicly available through the Figshare repository^75^, and a live version with potential updates is available in the GitHub repository (https://github.com/0oshowero0/HealthyCitiesDataset). To facilitate data access and utilization, we organise the dataset into several subsections (see Fig. 1). Specifically, the samples of life expectancy data, physical & mental health data, basic statistics data, behavioural environment data, built environment data, natural environment data and health outcomes data are demonstrated in Tables 5–10 accordingly. It provides convenience to researchers who only hope to access part of the data by reducing the data loading time.

**Table 5:** Example of life expectancy of the produced dataset.

Geographical Key		Life Expectancy		
MSOA Name	MSOA Code	Life Expectancy	Healthy Life Expectancy	Gender
Birmingham 008	E02001834	79.0 (76.7–81.3)	58.7 (57.1–60.3)	Male
Birmingham 011	E02001837	80.9 (79.2–82.6)	54.4 (53.2–55.5)	Female
Bradford 017	E02002199	81.3 (79.4–83.1)	67.3 (65.9–68.7)	Male
Bradford 019	E02002201	80.6 (79.1–82.0)	62.6 (61.5–63.8)	Female
…	…	…	…	…

^1^Numbers in parentheses indicate 95% confidence intervals.

**Table 6:** Example of physical & mental health of the produced dataset.

Geographical Key		Physical Health								Mental Health
MSOA Name	MSOA Code	Asthma	Cancer	Dementia	Diabetes	Hyperlipidemia	Hypertension	Obesity	COVID	
Birmingham 008	E02001834	2.33	0.0458	0.0378	4.48	0.645	1.78	0.0694	4	1.12
Birmingham 011	E02001837	0.451	0.00496	0.00748	0.564	0.0644	0.240	0.0309	4	0.176
Bradford 019	E02002201	1.27	0.0208	0.0760	1.427	0.261	0.944	0.0160	5	0.733
Bradford 024	E02002206	1.12	0.0238	0.0818	1.408	0.158	0.826	0.00854	3	0.449
…	…	…	…	……	…	…	…	…	…	…

^1^COVID data is available on a weekly basis. All other data are available on a monthly basis.

^2^COVID-19 data represents new cases by Specimen date calculated in a 7-day rolling window.

**Table 7:** Example of basic statistics subsection of the produced dataset.

Geographical Key		Basic Statistics				
MSOA Name	MSOA Code	Population	Area	Population Density	Geographical Centroid	Boundary
Birmingham 008	E02001834	6002	1.07	5622	(−1.89225,52.55562)	(−1.88401 52.55796, −1.88347 52.55703…)
Birmingham 011	E02001837	10327	1.48	6963	(−1.87804,52.54658)	(−1.86724 52.55160, −1.86930 52.55064…)
Bradford 017	E02002199	6891	8.92	773	(−1.72114,53.85007)	(−1.71462 53.86599, −1.71029 53.86349…)
Bradford 019	E02002201	12244	2.72	4502	(−1.73013,53.83329)	(−1.73784 53.84000, −1.73931 53.83876…)
…	…	…	…	…	…	…

^1^Geographical centroid and boundary are available in WGS84.

**Table 8:** Example of behaviour environment subsection of the produced dataset.

Geographical Key		Behaviour Environment			
MSOA Name	MSOA Code	Tobacco Availability	Alcohol Availability	Health Service Availability	Physical Exercise Availability
Birmingham 008	E02001834	0.00100	0.00133	0.00150	0.0000968
Birmingham 011	E02001837	0.000484	0.00107	0.00126	0.000157
Bradford 017	E02002199	0.000290	0.00145	0.000580	0
Bradford 019	E02002201	0.000490	0.000904	0.00310	0
…	…	…	…	…	…

**Table 9:** Example of built environment subsection of the produced dataset.

Geographical Key		Built Environment					
MSOA Name	MSOA Code	Building Density	Median/Mean House Price	Driving/Cycling/ Walking Road Density	Street View Features	Satellite View Features	Walkability
Birmingham 008	E02001834	2332	177000/180152	24.0/24.0/24.5	0.101/0.0693/…	0.171/0.110/…	1.48
Birmingham 011	E02001837	2614	161000/164506	17.4/18.1/20.7	0.0768/0.0660/…	0.151/0.0811/…	0.953
Bradford 017	E02002199	70	233725/253598	7.02/10.1/13.4	0.0785/0.0396/…	0.128/0.0624/…	−0.885
Bradford 019	E02002201	201	152500/181140	21.2/22.8/31.7	0.0764/0.0354/…	0.157/0.0689/…	−0.202
…	…	…	…	…	…	…	…

^1^Median/Mean house prices are available on a quarterly basis.

^2^Street view features consist of 19 columns, as demonstrated in Table 4.

^3^Satellite features consist of 7 columns, as demonstrated in Table 4.

**Table 10:** Example of natural environment subsection of the produced dataset.

Geographical Key		Natural Environment						
MSOA Name	MSOA Code	NO_x_/PM2.5/ PM10	Min/Max Temperature	Rainfall	Relative Humidity	Snow Lying Days	Sunshine Hours	Wind Speed
Birmingham 008	E02001834	45/8/13	5.98/9.32	0.0231	83.7	2.18	46.6	3.60
Birmingham 011	E02001837	45/8/13	6.13/9.56	0.0176	83.4	1.72	47.0	3.36
Bristol 001	E02003012	41/12/15	6.27/10.6	0.000064	82.7	0.00	55.4	3.95
Bristol 044	E02003055	41/12/15	5.90/10.9	0.000046	83.2	0.00	53.9	3.00
…	…	…	…	…	…	…	…	…

^1^The air quality data of NO_x_/PM2.5/PM10 contain multiple records for observations from different stations.

^2^NO_x_/PM2.5/PM10, min/max temperature and rainfall data are available on a daily basis.

^3^Relative humidity, snow lying days, sunshine hours and wind speed are available on a monthly basis.

All the data are available in tabular format, where the MSOA codes or city codes are used to correlate different subsections of the data. We provide the geographic lookup table demonstrated in Table 3 for users who are interested in larger geographical scales such as LTLA or UTLA level. For the life expectancy data in Table 5, we provide gender-specific life expectancy and healthy life expectancy with 95% confidence intervals. For the physical health and mental health data in Table 6, we provide monthly expenditures per citizen for asthma, cancer, dementia, diabetes, hyperlipidemia, hypertension, obesity, and general mental disorders. For the COVID-19 data, we provide new cases time series in a 7-day rolling window, which is available on a weekly basis. For the basic statistics in Table 7, we have population, area size, population density, geographical centroid, and boundary polygon information. The area size is available in km^2^, and the centroid data and boundary are available in WGS84. The behaviour environment in Table 8 contains the availability of tobacco, alcohol, health service, and physical exercise POIs by the corresponding POI number divided by the population size. For the built environment in Table 9, we provide building density, median/mean house price, driving/cycling/walking road density, street view features, satellite features, and walkability score. The building density represents the number of buildings per km^2^, and the house price data are available on a quarterly basis. The road density data represents the average road length (in *km*) per *km*^2^. The street view and satellite view features demonstrate the average percentage of each visual element in the image data. Walkability represents the average z-score of population density, intersection density and daily living score. For the natural environment in Table 10, we provide the NO_x_, PM2.5, PM10 indices (*ug/m*^3^), min/max temperature (°C), rainfall (*mm*), relative humidity (%), snow lying days (days per month), sunshine hours (hours per month), and wind speed (knots). Except for the snow lying days, sunshine hours and wind speed that are available in a monthly basis, all other natural environment data are available on a daily basis.

Intensive correlations between environmental factors and health outcomes can be discovered through the data records. For instance, the availability of bars is linked with alcohol-related harms^22,23^, which can be evaluated through the alcohol availability in Table 8 with the drug expenditure in Table 6. Recent studies also demonstrate that street view images are predictive for COVID-19 infections, obesity, diabetes, mental distress, etc.^53,54^, which can be evaluated through the street view features in Table 9. Besides, researchers can also validate the building and road densities in Table 9 with dementia expenditure to validate their influence on cognitive function^41,76^. For the natural environment data in Table 10, we can correlate the air pollution data with the expenditure for mental disorders in Table 6 to validate the effect on psychopathology^65^, or investigate the influence of temperature and other weather features for citizens’ health^72,73^. Furthermore, our data provide an opportunity to investigate the high-order correlation between various environmental factors and health outcomes, which is still an unresolved research question. Consequently, our produced data will benefit and facilitate a plethora of related studies.

## Technical Validation

### Representativeness of selected cities

**Fig. 3:**
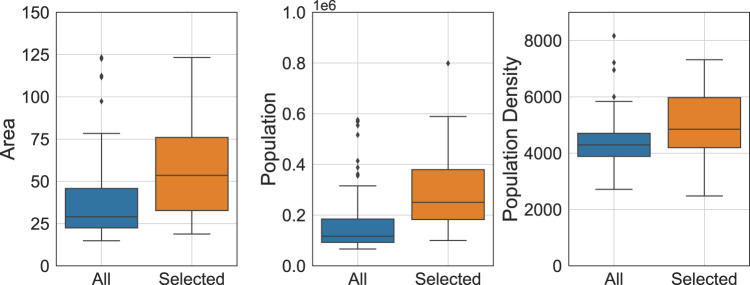
Representativeness of selected cities in our dataset. The box demonstrates the median and quartiles of the data, and the whiskers extend represent the rest of the distribution (except the outliers).

In this study, we select representative England cities according to the availability of the source data, where cities that have high status in economic, political and cultural perspectives have been included in our dataset. In Fig. 3, we demonstrate the distribution of area, population and population density of selected cities and all major towns and cities according to ONS^29^. We find that the selected cities are able to cover most of the area and population ranges of all the major towns and cities.

### Feature extraction of image data

**Fig. 4:**
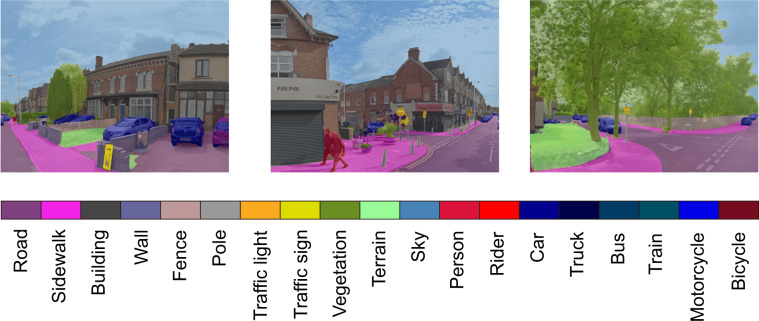
Example of semantic segmentation results for street view images in Birmingham.

**Fig. 5:**
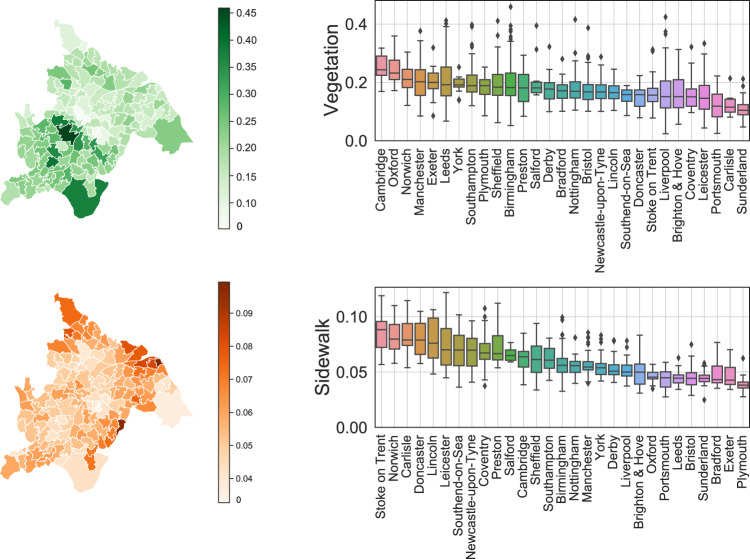
Visualization of extracted features from street view images. We demonstrate the MSOA level vegetation and sidewalk indicators in Birmingham, and city level distribution in all city-of-interests. The box demonstrates the median and quartiles of the data, and the whiskers extend represent the rest of the distribution (except the outliers).

**Fig. 6:**
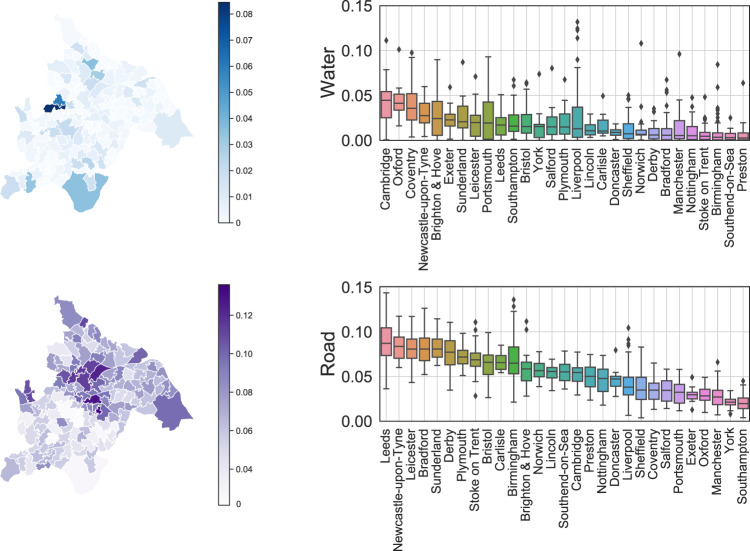
Visualization of extracted features from satellite view images. We demonstrate the MSOA level water and road indicators in Birmingham, and city level distribution in all city-of-interests. The box demonstrates the median and quartiles of the data, and the whiskers extend represent the rest of the distribution (except the outliers).

We adopt the deep learning model to mine the semantic information in both street view and satellite view imagery. To ensure the reliability of the generated features, several quality control procedures are adopted. First, we choose the state-of-the-art deep learning model that ranks first for unseen images in the segmentation task. The standardized benchmarks in the computer vision community ensure the reliability of model selection. Specifically, we use the ViT-Adapter model^55^ for both street view and satellite view images, which leverages the recent advances in vision transformer^77^ to greatly improve the accuracy and generalizability of semantic segmentation models. Second, we select the training dataset that includes varying scenarios to enhance the transferability of the model. For street view image segmentation, we use the Cityscapes dataset^57^, which is one of the standard datasets for segmentation tasks. It contains 25000 annotated urban street scenes for 50 different cities in a variety of seasons, daytime, and weather conditions. For satellite view segmentation, we choose the famous LoveDA dataset^61^ that contains 5987 high spatial resolution satellite images for 18 different administrative districts in both urban and rural areas. The wide coverage of training data helps the model to provide reliable results and ensures a successful transfer to images of the UK, which is shown in Fig. 4. Third, for hyperparameters of the model, we use the official implementation provided by the author of ViT-Adapter, where extensive parameter searching and training tricks have been done to make the model rank first. Specifically, AdamW optimizer with an initial learning rate of 2*e*−5 and weight decay of 0.05 is used to train the model. The full hyperparameter table can be found through the GitHub repository^78^. Through these parameter combinations, the inference performance achieves a high all pixel accuracy (aAcc) of 97.02% and mean intersection over union (mIoU) of 84.46% for unseen street view images. For the satellite view images, we achieve high performance with aAcc of 71.11% and mIoU of 52.73%, surpassing the state-of-the-art model with mIoU of 52.44%^79^. Fourth, we further examine the extracted features by human experts to preclude possible defects. For street view segmentation, we visualize the calculated features of vegetation and sidewalk from both the MSOA level and city level and provide an example in Fig. 5. For the satellite view segmentation, we showcase the extracted water and road percentage in Fig. 6.

### Experiments to examine environmental factors with health outcomes

**Fig. 7:**
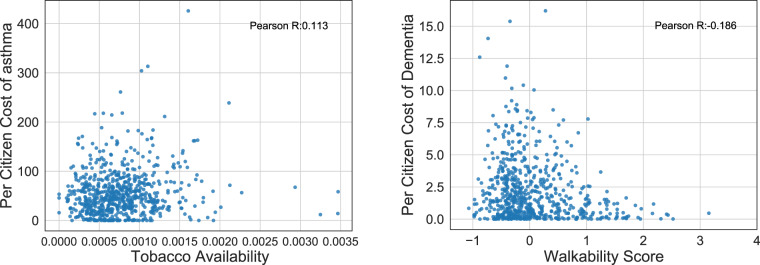
Examples of simple correlation test between environmental factors with health.

We design some experiments to verify the proposed environmental factors with health outcomes in our dataset, according to the intuition from public health literature. Specifically, the smoking behaviour is positively correlated with asthma incidence^80–82^, while the walking behaviour is negatively correlated with dementia^76,83,84^. Through our dataset, we use the availability of tobacco POI as the agent for regional level tobacco usage, and the walkability score for walking behaviour. We demonstrate the relationship between the above environmental features with per citizen asthma and dementia expenditure in Fig. 7. We observe a positive correlation of 0.113 for tobacco availability and the cost of asthma, and a negative correlation of −0.186 for walkability and dementia. These observations are consistent with the existing studies, validating the effectiveness of the produced dataset.

## Usage Notes

There are several limitations in the present work. First, the definition of “environment” can be broad: from the concrete concept of neighbourhoods for daily living^53^, to the abstract social and cultural atmosphere^85^, all these environments could affect public health. Considering the wide range of associations between health and other factors, we focus on physical environments and their health outcomes as quantifiable indicators, while the impact of other factors might also affect the health outcomes summarized in our work. Second, limited by the varying sample frequency of the raw data, we cannot merge the data into a unified time resolution. For instance, the temperature data is daily updated, while the house price is summarized quarterly. Therefore, researchers should be aware that the temporal differences between data records might affect their findings. Third, we use pre-trained semantic segmentation models on standard benchmarks (*e.g*., Cityscapes) to extract the imagery features from street view and satellite images in the UK, where the accuracy might fluctuate due to the generalizability of the deep learning method. By carefully choosing training benchmarks with high diversity and validating the extracted features, the semantic segmentation models provide reasonable results on UK images and ensure the reliability of the dataset. Researchers should be aware of the scope and limitations of our dataset to make informed judgements on the relationship between environmental determinants and public health.

## Data Availability

The Python codes to generate the dataset are publicly available through the GitHub repository (https://github.com/0oshowero0/HealthyCities). Detailed instruction for software environment preparation, folder structure and commands to run the provided codes is available in the repository.
